# Spring Is Coming: Genetic Analyses of the Bud Break Date Locus Reveal Candidate Genes From the Cold Perception Pathway to Dormancy Release in Apple (*Malus* × *domestica* Borkh.)

**DOI:** 10.3389/fpls.2019.00033

**Published:** 2019-03-07

**Authors:** Yohanna Evelyn Miotto, Carolina Tessele, Ana Beatriz Costa Czermainski, Diogo Denardi Porto, Vítor da Silveira Falavigna, Tiago Sartor, Amanda Malvessi Cattani, Carla Andrea Delatorre, Sérgio Amorim de Alencar, Orzenil Bonfim da Silva-Junior, Roberto Coiti Togawa, Marcos Mota do Carmo Costa, Georgios Joannis Pappas, Priscila Grynberg, Paulo Ricardo Dias de Oliveira, Marcus Vinícius Kvitschal, Frederico Denardi, Vanessa Buffon, Luís Fernando Revers

**Affiliations:** ^1^Department of Crop Science, Agronomy School, Federal University of Rio Grande do Sul, Porto Alegre, Brazil; ^2^Embrapa Uva e Vinho, Bento Gonçalves, Brazil; ^3^Graduate Program in Cell and Molecular Biology, Centro de Biotecnologia, Federal University of Rio Grande do Sul, Porto Alegre, Brazil; ^4^Programa de Pós-Graduação em Ciências Genômicas e Biotecnologia, Universidade Católica de Brasília, Brasília, Brazil; ^5^Embrapa Recursos Genéticos e Biotecnologia, Brasília, Brazil; ^6^Department of Cell Biology, Universidade de Brasília, Brasília, Brazil; ^7^Empresa de Pesquisa Agropecuária e Extensão Rural de Santa Catarina – Epagri – Estação Experimental de Caçador, Caçador, Brazil

**Keywords:** apple, bud dormancy, chilling requirement, linkage mapping, *MdoFLC*, *MdoICE1*, *MdoPRE1*

## Abstract

Chilling requirement (CR) for bud dormancy completion determines the time of bud break in apple (*Malus* × *domestica* Borkh.). The molecular control of bud dormancy is highly heritable, suggesting a strong genetic control of the trait. An available Infinium II SNP platform for genotyping containing 8,788 single nucleotide polymorphic markers was employed, and linkage maps were constructed in a F_1_ cross from the low CR M13/91 and the moderate CR cv. Fred Hough. These maps were used to identify quantitative trait loci (QTL) for bud break date as a trait related to dormancy release. A major QTL for bud break was detected at the beginning of linkage group 9 (LG9). This QTL remained stable during seven seasons in two different growing sites. To increase mapping efficiency in detecting contributing genes underlying this QTL, 182 additional SNP markers located at the locus for bud break were used. Combining linkage mapping and structural characterization of the region, the high proportion of the phenotypic variance in the trait explained by the QTL is related to the coincident positioning of Arabidopsis orthologs for *ICE1, FLC*, and *PRE1* protein-coding genes. The proximity of these genes from the most explanatory markers of this QTL for bud break suggests potential genetic additive effects, reinforcing the hypothesis of inter-dependent mechanisms controlling dormancy induction and release in apple trees.

## Introduction

The domesticated apple (*Malus* × *domestica* Borkh.), as other temperate fruit trees, has developed the ability to enter a dormant state, which is a mechanism that enables plants to survive seasonal changes and protect sensitive meristems from unfavorable climatic conditions (Rohde and Bhalerao, 2007). Bud dormancy is usually divided into three consecutive phases: para-, endo-, and ecodormancy, where budburst inhibition is caused by signals derived from outside of the bud (but from the same plant), from the bud itself, or from environmental conditions, respectively (Lang, 1987). Endodormancy establishment is characterized by growth cessation, bud set, and leaf senescence, and the process is gradually overcome by prolonged exposure to cold. Each species and/or cultivar need a certain amount of low temperature exposure called chilling requirement (CR), in order to remove the physiological blocks that inhibit budburst and culminates into ecodormancy. In the ecodormant phase, the meristems achieve full developmental capacity and resume growth after a certain amount of warmth, which is also genotype-dependent (Dennis, 2003; Legave et al., 2008; Fu et al., 2014).

The physiological aspects of dormancy control have been partially elucidated and there are evidences that the mechanisms regulating dormancy release are highly heritable, suggesting a strong genetic control (reviewed in Falavigna et al., 2015). In fruit trees, the comprehension of molecular control of dormancy has been improved by the study of the *evergrowing* (*evg*) peach mutant, which fails to enter dormancy and maintains constant growth (Bielenberg et al., 2008). Mapping of the *evg* locus revealed six tandemly arranged MADS-box genes that were named *Dormancy-Associated MADS-box* (*DAM*). Further, functional characterization of some Rosaceous *DAM* orthologs proved their importance in the control of bud dormancy (Sasaki et al., 2011; Yamane and Tao, 2015; Wu et al., 2017). In apple, different strategies have been explored so far in order to understand the genetics of bud break date (BBD) and flowering time. Employing linkage map analysis on a bi-parental family, the first two quantitative trait loci (QTLs) for time of bud break were identified (Segura et al., 2007). In a similar approach, the end of the linkage group 9 (LG9) has been identified as a major QTL for time of bud break and flowering in other four progenies (van Dyk et al., 2010; Celton et al., 2011). Using a multi-parental population and pedigree-based analysis, Allard et al. (2016) detected a strong QTL for bud break and flowering time in the same chromosomal position, as well as another five QTLs of small effect coincident with the position of the *DAM* genes, the florigen *FLOWERING LOCUS T* (*FT*), or the flowering repressor *FLOWERING LOCUS C* (*FLC*). More recently, a genome-wide association (GWAS) study explored an apple core collection and confirmed the position of the major QTL for time of bud break on LG9, besides narrowing the confidence interval to ∼360 kb and emphasizing the importance of two major candidate genes encoding transcription factors containing NAC or MADS-box (putative *FLC*) domains (Urrestarazu et al., 2017).

In Arabidopsis, floral initiation can be induced by long-term chilling exposure, which is able to trigger the epigenetic suppression of *FLC* in a process called vernalization (Hepworth and Dean, 2015). Although the vernalization process somewhat resembles dormancy in temperate plants, the *FLC* role in perennial plants is still not fully understood. In apple, a putative *FLC* ortholog was identified and had a proposed role in dormancy release (ecodormancy) based on its differential expression during dormancy along with its co-localization in a QTL related to bud break (Porto et al., 2015). One of the positive regulators of Arabidopsis *FLC* expression is the CBF/DREB (C-repeat binding factor/dehydration-responsive element-binding protein) class of transcription factors, which are major integrators of plant cold response (Seo et al., 2009; Thomashow, 2010). Interestingly, ectopic expression of the peach *CBF1* gene in apple rendered increased freezing tolerance, early bud set and leaf senescence, and delayed bud break (Wisniewski et al., 2015). *CBF* gene expression is under direct regulation of ICE1 (Inducer of CBF Expression 1), a MYC-like basic-helix-loop-helix (bHLH) transcription factor that acts as a convergence point integrating cold and several other signaling pathways (Ding et al., 2015). Higher expression of a putative *ICE1* gene was found during pear endodormancy stage suggesting that it is importance in endormancy maintenance (Takemura et al., 2015). Additionally, an apple *ICE1* gene has been previously identified as differentially expressed during dormancy when comparing contrasting CR cultivars (Falavigna et al., 2014). These evidences strongly suggest that the cold sensing pathway might be acting during bud dormancy in apple. The same strategy employed to identify apple *FLC*, i.e., differential gene expression and co-localization in a bud break QTL, also yielded the identification of another gene that might play a role during apple dormancy, *PACLOBUTRAZOL RESISTANCE 1* (*PRE1*; Porto et al., 2015). As ICE1, PRE1 is a bHLH transcription factor, and its role is to integrate signals from brassinosteroids, gibberellin (GA), and light pathways. In addition, *PRE1* overexpression renders early flowering in Arabidopsis (Lee et al., 2006; Bai et al., 2012).

Despite of the significant advances, the genetic mechanisms of dormancy induction and release in apple trees are not fully understood. Therefore, it is still necessary to continue exploring the biological relevance of beginning of chromosome 9 in dormancy regulation. Within this context, the purpose of this study is to better characterize the LG9 QTL for BBD, a trait directly linked to CR and flowering, by enriching the LG9 with a set of newly developed SNPs for genotyping. Combining linkage mapping and a detailed structural characterization of the beginning of chromosome 9, we confirmed the position of the major QTL for BBD in LG9 and identified a new highly relevant candidate gene, the cold perception gene *MdoICE1*. The identification of *MdoICE1*, along with the already known *MdoFLC* and *MdoPRE1* genes previously identified by our group (Porto et al., 2015), provides us clues to propose that these genes are fully integrated in pathways leading from cold perception to responses associated to growth resumption after dormancy fulfillment. We postulate that these three genes may have additive effects on dormancy-associated phenotypes, reinforcing the hypothesis of inter-dependent mechanisms controlling dormancy induction and release in apple trees.

## Materials and Methods

### Plant Materials

The plant material in our study consisted of one full sib (FS) family, including a total of 190 progeny individuals. This FS family (mapping population) was derived from a controlled pollination F1 cross between the low CR “M13/91” and the moderate CR “Fred Hough.” The parental germplasm and the FS family were developed by Epagri – Caçador Experimental Station (Santa Catarina, Brazil), grafted on the widely used clonal apple rootstock M7 and planted in 2010 in two locations: 161 individuals were planted in a site located in Bento Gonçalves (BG, Rio Grande do Sul, Brazil; 29° 09′S, 51° 31′W; altitude 623 m) and 114 individuals in Vacaria (VC, Rio Grande do Sul, Brazil; 28° 33′S, 50° 57′W; altitude 970 m), Southern Brazil. The two site locations have distinct climate conditions that provide contrasting scenarios for the phenotypic segregation of CR and related traits. VC represents the condition of the Brazilian commercial orchards (type Cfb, Alvares et al., 2014) and BG as the condition of more mild/warm winters (type Cfa; Alvares et al., 2014; Supplementary Tables S1, S2).

### Phenotyping

The main phenological stage evaluated was vegetative BBD (Bud Break Date). The phenotypic value of the trait corresponds to the date of the green point in the Fleckinger’s apple phenological classification (EPPO, 1984). Phenotyping trait assessments were performed three times a week (day 1 being July 1st) over a period of seven growing seasons in BG (2011–2017) and five growing seasons in VC (2011, 2012, 2014, 2015, and 2017). Phenotypic and genotypic association was tested employing a chi-square independency test, separately for planting sites and year of growing cycle.

### SNP Genotyping

#### IRSC (International Rosaceae SNP Consortium) SNP Markers

DNA from the parents and the FS family were extracted from samples of young leaf tissue, following Lefort and Douglas (1999) method modified to fit 2 mL microcentrifuge tubes. The DNA quality and concentration was checked using a NanoDrop 2000 spectrophotometer (Thermo Scientific). Genomic DNA from samples of the individuals was amplified with no allelic partiality, followed by enzymatic fragmentation, precipitation, and DNA resuspension accordingly to Infinium II whole-genome genotyping assay protocol (Illumina, Inc., San Diego, CA, United States). Samples were applied to BeadChips containing sequence probes to interrogate 8,788 SNP positions distributed across the “Golden Delicious” apple genome assembly, *Malus* × *domestica* v1.0 (Velasco et al., 2010). These SNP makers were developed by the IRSC SNP Consortium as described previously (IRSC 9k Apple SNP array; Chagné et al., 2012). SNP genotyping was performed at the Genotyping and Genetic Diagnosis Unit (University of Florida, Gainesville, FL, United States). SNP genotypic data analysis and quality control were performed using GenomeStudio V2011.1 pipeline (Illumina, Inc.) using a SNP call rate filtering threshold of 0.90.

#### Kompetitive Allele Specific PCR (KASP) SNP Markers and Genotyping

To increase the SNP density for our QTL mapping study, we developed a new set of SNP markers. Whole-genome sequencing was performed using DNA samples from four individuals of “M13/91” (2) and “Fred Hough” (2). Sequencing was made using Illumina GA II platform, 1 × 100 bp, and generated 38 million reads (average coverage 6×) and 60 million reads (average coverage 10×), respectively, with reliable mapping on the genome assembly of “Golden Delicious,” *Malus* × *domestica* v1.0 (Velasco et al., 2010). The data set of sequences generated was deposited under the access number PRJNA479978. With this sequence data, a large number of SNPs shared by the plant lines were detected (Alencar et al., 2011). These SNPs were further inspected for the development of genotyping assays targeting high-density mapping of apple LG9 based on the integration of the apple genetic map (Muranty et al., 2014) with contigs in the apple genome sequence. SNP positions deemed polymorphic between the two plant cultivars and located within predicted exons in the gene content annotation were considered primary targets for the design of genotyping assay using KASP technology. To provide enough sequence flank, we extracted a 70-bp string of nucleotides toward each direction from the target SNP using BedTools (Quinlan and Hall, 2010) and the reference genome. The set of sequences with size of 141 bp each were screened for features that could compromise the genotyping such as low complexity stretches of DNA and the presence of small units of repeats using MISA (Beier et al., 2017). The final set of 182 sequences containing putative new SNPs was used as source to the design of KASP markers for genotyping (Supplementary Table S3). Leaf discs with 10 mm diameter were freeze-dried and genotyped using the KASP technique at LGC genomics (Teddington, Middlesex, United Kingdom). Homozygous SNPs for both parents were discarded.

### Genetic Linkage Map Construction and QTL Analysis

Genetic mapping of the parents was performed using JoinMap^®^4 (Van Ooijen, 2006). Segregation distortion of individual markers was revealed by chi-square test. Markers showing distorted segregation (*P* < 0.05) were removed from analysis. Linkage analysis was performed using the regression approach implemented for cross-pollinator (CP) with a minimum logarithm of the odds (LOD score) of >8 used to define LG. The Kosambi mapping function was applied for map distance calculation (Kosambi, 1944). LGs were numbered in accordance with Maliepaard et al. (1998) and graphs were prepared using MapChart 2.2 (Voorrips, 2002).

Quantitative trait loci analysis was performed with MapQTL^®^6 (Van Ooijen, 2009) using the phenotypic traits assessment from BG and VC. Regions with potential QTL effects were identified using interval mapping (IM) and restricted multiple QTL mapping (rMQM) functions. QTLs were declared significant if the maximum LOD, obtained after multiple rounds of rMQM mapping, exceeded the LG and genome-wide (GW) LOD threshold (calculated with 1,000 permutations, mean error rate of 0.05). QTLs were characterized by the maximum LOD score and percentage of phenotypic variation explained. Broad sense heritability (*H*^2^) for BBD was estimated based on the components of variance from the phenotypic analysis for both. Analyses were performed using the GLM procedure (Proc GLM) of the statistical software SAS/STAT^®^ software (SAS Institute Inc., 2008).

### *In silico* Candidate Gene Search

We listed the predicted gene structures in the contigs containing the markers of potential QTL effects in the chromosome 9 contributing to the trait value differences in the F1 cross. Gene Ontology terms for these genes were collected from the annotations in the genome assembly of “Golden Delicious,” *Malus* × *domestica* v1.0, downloaded from PLAZA v2.5 platform (Van Bel et al., 2012). The gene list and the corresponding ontology terms were used to test if genes that belong to one ontology term do not differ in their ranks from genes that do not belong to that term, namely, the null hypothesis. The average LOD score for each marker in the MapQTL6 analysis using the phenotypic traits assessment from site locations BG and VC was used as the associated variable to rank the genes. Taken this continuous variable as the likelihood ratio of map a gene to the BBD QTL, we computed the Wilcoxon rank sum statistic, which is the sum of the ranks of the ontology term as a group for all corresponding genes in the whole chromosome. Approximate *p*-values for the rank test were obtained based on the method implemented in the program *func_wilcoxon* in the FUNC package (Prüfer et al., 2007). Significance of the tests was assessed in terms of a global *p*-value derived using a sensitive estimator in detecting a deviation from the null hypothesis. We used a cut-off of 0.05 for the global *p*-value to detect ontology terms that differs in their ranks. *P*-values declaring significance for one side of the test statistic were used to detect enrichment of gene-associated variables among the terms. Markers in the genes containing ontology terms that show enrichment were considered anchors to delimitate candidate regions of the BBD QTL in the chromosome 9 for *in silico* candidate gene search.

### Physical Mapping of SNPs to the Assembly of a “Golden Delicious” Doubled-Haploid Tree (GDDH13) and Characterization of Candidate Regions of the BBD QTL

The positions of the SNP markers used in genotyping were physically mapped on the sequence of the pseudomolecule in the recently published genome assembly of Daccord et al. (2017). Sequences for the pseudomolecules were retrieved from the apple genome and epigenome project website (^1^GDDH13 v1.1). As the SNPs are originally described using a previous assembly (*Malus* × *domestica* v1.0), we mapped the v1.0 SNPs to the GDDH13 genome following UCSC same species lift over procedure^2^. In summary, UCSC software tools (Kuhn et al., 2013) were used to create the alignment chain file between the two assemblies, and then we used CrossMap (Zhao et al., 2014) to convert the coordinates of the SNPs to the GDDH13 assembly. Using the coordinates of the markers highlighted in the QTL and Gene Ontology analyses, we defined candidate regions of the BBD QTL to perform careful inspection for prioritizing candidate genes (see section “Results” for the criteria utilized). We extracted and summarized functional annotation associated with individual genes and with group of genes with related ontology terms. Additionally, we performed scans of the sequences in the 5′ flanking sequence of the individual genes to define *cis*-acting elements putatively involved to chilling response in plants. PlantPAN 2.0 (Chow et al., 2016) database and tools were used for all the plant promoter analysis.

## Results

### Phenotypic Trait Assessment

**FIGURE 1:**
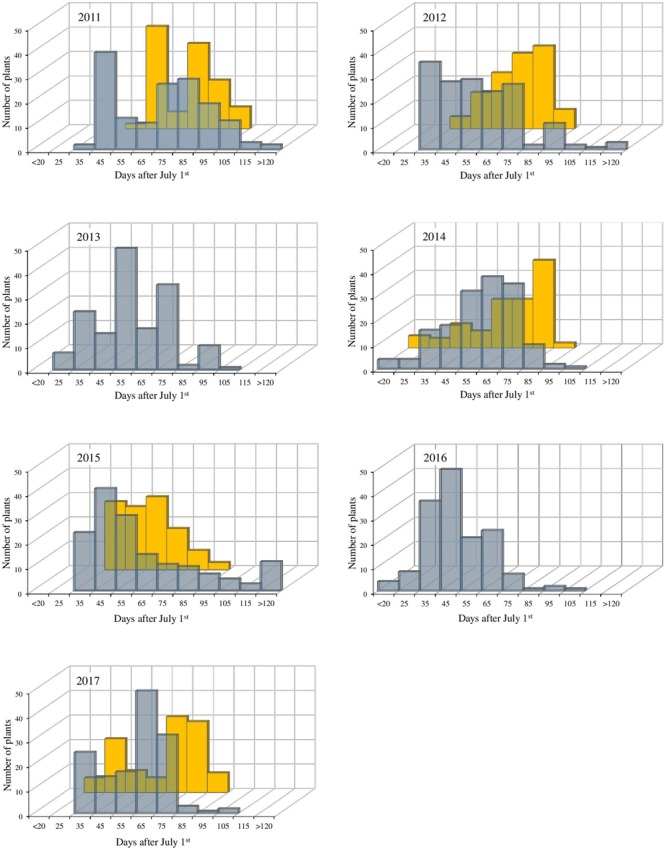
Distribution of the bud break date (in days, starting from July 1st) for the population “M13/91” × “Fred Hough” in the evaluated years. Gray bars represent the experimental site in BG and yellow bars represent the experimental site in VC.

Apple BBD is mostly regulated by exposure to low temperatures (Heide and Prestrud, 2005). Therefore, we decide to use BBD measurements as the main indicator for CR in the mapping population. The observed BBD for the individuals in each year and at both sides showed remarkable differences and provided insights on genotype and environment interactions (Figure 1). Large phenotypic variation was observed between both sites, and BBD window was generally earlier and wider in BG (mean of observed extremes: 25 to 118 days) when compared to VC (mean of observed extremes: 32 to 87 days). Despite of this trait variability, the estimated broad sense heritability (*H*^2^) was consistently high (*H*^2^ = 0.54 in BG and 0.73 in VC), reinforcing the strong genetic effect for bud phenology traits linked to CR.

### Linkage Mapping Construction

The F_1_ progeny was genotyped using the IRSC 9k Infinium II array (Chagné et al., 2012). In total, 2,733 SNP markers (30.37%) passed the SNP calling and filtering from the GenomeStudio V2011.1 pipeline (Illumina, Inc.) and were used for linkage analysis. From the 182 KASP-SNPs previously selected for the LG9 high density mapping, 114 generated polymorphic amplification, and were further used, totaling 2,847 genotyped SNP markers. Following the double pseudo-testcross strategy, marker sets from either parent were separately processed for both maps. The genetic linkage map constructed for the parental “M13/91” covered 1,293.77 cM and consisted of 784 markers (Supplementary Table S4). Out of the 114 KASP-SNP markers tested, 34 markers were successfully integrated into the LG9 end (Supplementary Figure S1). LG10 was the longest LG on the map, spanning 101.68 cM, while LG17 was the shortest, spanning 32.42 cM. The highest number of markers mapped to a single LG was 75 on LG2, the smallest number of markers on a single LG was 10 on LG17 (Supplementary Figure S1 and Supplementary Table S4). The map had an average marker density of one marker every 1.99 cM. The linkage map contained 18 regions in excess of 10 cM that contained no mapped molecular markers.

The genetic linkage map constructed for the parental “Fred Hough” covered 1,013.99 cM and consisted of 791 markers. Thirty-four KASP-SNP markers were successfully integrated in LG9 (Supplementary Figure S1). LG3 was the longest LG on the map, spanning 70.84 cM while LG11 was the shortest, spanning 27.52 cM. The highest number of markers mapped to a single LG was 91 on LG3 and the smallest number was 16 markers on LG11 (Supplementary Figure S1 and Supplementary Table S4). The map had an average marker density of one marker every 1.55 cM and enclosed a total of nine regions in excess of 10 cM that contained no mapped molecular markers. Parental maps were aligned using common markers (Supplementary Figure S1) and exhibited 17 LGs corresponding to the number of chromosomes in the apple genome assembly (Velasco et al., 2010; Daccord et al., 2017). The order of the markers on the genetic maps was thoroughly checked, and their correspondence to the apple doubled haploid genome was performed (Daccord et al., 2017). Overall, the SNP marker order was coherent. However, the following main issues were identified that are common to other reports in the literature: (i) inconsistencies in LG assignment for 17% of the markers; (ii) regions of inversion (max ∼ 2.5 Mbp) involving 3% of the markers; and (iii) misplaced regions of markers within the same pseudo-chromosome for 2% of the markers.

### QTL Detection

**FIGURE 2:**
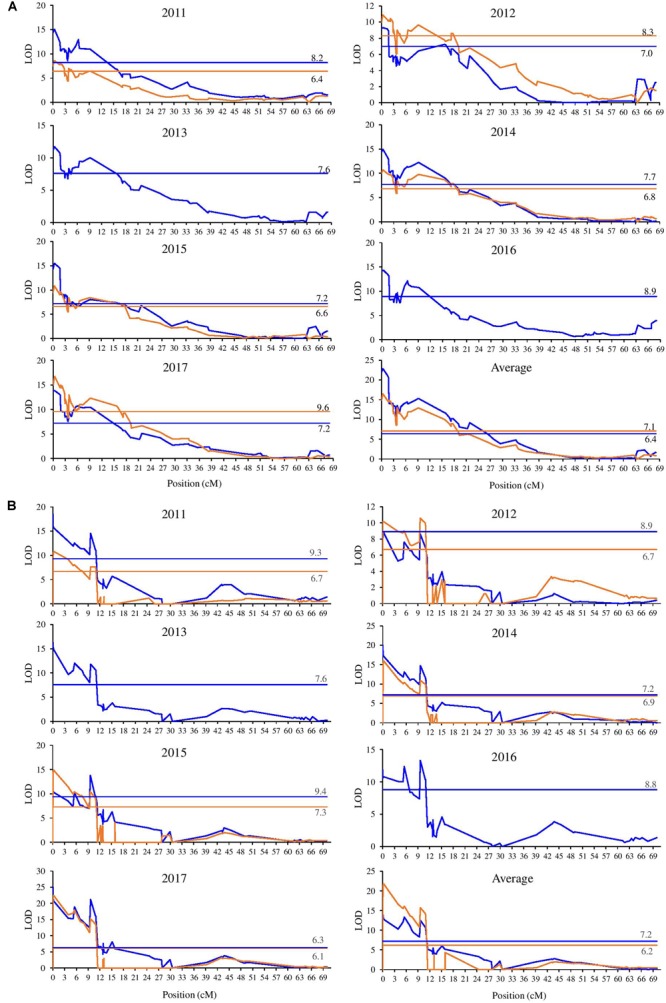
Position of the QTL for bud break date detected in LG9 of the “M13/91” × “Fred Hough” maps. (Blue lines represent BG and orange lines represent VC. The threshold values are indicated.) **(A)** Fred Hough. **(B)** M13/91.

Quantitative trait loci analysis was performed separately on the parental maps for each set of phenotypical data obtained (BG and VC), and are shown in Supplementary Table S5 and Figure 2. In all analyzed years, a single QTL region for BBD was detected for both parents at the beginning of LG9. Analysis performed on the BBD data for the two different population sites showed that this QTL exceeded the LG and GW LOD thresholds for all evaluated years. The BBD QTL located at the LG9 explained 24.5–45.6% of the phenotypic variance in BG population and 31–48% in VC population across different years (Supplementary Table S5). This result strongly suggests that a major effect locus contributing to trait value differences of vegetative BBD is located at the beginning of LG9. Overall, the LODs distribution across mapped intervals in the parental maps are reasonable conserved over years and sites, showing always a peak at the extremity of the LG9 on both parents. The distribution of significant LODs for the BBD QTL on “M13/91” LG9 was stable and restricted to the first 10–11 cM (Supplementary Table S5) when compared to the “Fred Hough” progenitor, which spanned a wider and more variable region. Examining the BBD QTL on “M13/91” progenitor, depending on the year and the LOD threshold, two remarkable peak regions are observed: the first at the extremity (marker TC_465054_Lg9) and the second starting around 9–11 cM (markers drm_qtl_snprnt113 and drm_qtl_snprnt137).

KASP-based SNP genotyping increased the number of markers anchored at the end of LG9. In M13/91, 10 exclusive markers were mapped in this region, spanning 9.3 cM more toward the end when compared to “Fred Hough.” This shift is also clearly visible in the LOD and phenotypic variance distribution observed in all evaluated years (Supplementary Table S5). When overlapping the LG9 BBD QTL on “M13/91” and “Fred Hough” linkage maps, it is observed that the “Fred Hough” BBD QTL starts at nearly the marker position where the “M13/91” BBD QTL approximately ends (see markers drm_qtl_snprnt113 and drm_qtl_snprnt137; Supplementary Table S5). These observations were used to define the region considered for detailed inspection in the search for candidate genes.

In order to demonstrate the markers contribution to the phenotypic variation for BBD, the top 15 markers associated with the QTL interval detected for both parents were tested. All the tested markers segregated for BBD in both sites (Supplementary Figure S2 and Supplementary Table S6). Remarkably, the heterozygous markers for the “M13/91” parent, showed clear phenotypic distribution, with significant value of chi-square calculated in BG and VC. The separation of individuals into three distinct groups, early, intermediate, and late BBD, highlights the association between “low CR alleles” and the precocity of the growth resumption in this population.

### *In silico* Candidate Gene Identification

We mapped the SNPs used in genotyping to the recently published high-quality doubled haploid genome assembly (Daccord et al., 2017). The distribution of the significant LODs in the QTL analysis for variation in the evaluated phenotype for both parents was fully investigated using the new coordinates. Markers that passed the LOD thresholds (LG and GW) for each parent were selected (Supplementary Table S5 and Figure 2). Taking into account the narrower LOD distribution, the stability over the years, the low chilling heritability and the mapping of 10 additional exclusive markers, the most likely segment of DNA (the locus) representing the BBD QTL detected for “M13/91” was selected.

Using the relevant markers, we performed gene ontology analysis on the base of Wilcoxon rank sum tests taken the significant LODs as gene-associated variables (Supplementary Table S7). Noteworthy, enriched terms for genes target by markers or in close proximity to them, i.e., located in the same contig, included transmembrane transport (GO:0055085) and anion transport (GO:0006820) in the biological process ontology, ATPase activity coupled to transmembrane movement of substances (GO:0042626) in the molecular function ontology, and integral component of membrane (GO:0016021) and mitochondrial outer membrane (GO:0005741) in the cellular component ontology. Sequence coordinates for the eight gene models highlighted in the analysis of differences of rank sum test for ontology terms (Supplementary Table S7) were adjusted to include 200,000 bp in each direction (Brodie et al., 2016). All book-ended (“touching”) entries were then merged into a single interval using, respectively, slopBed and mergeBed utilities from the BedTools v2.25 package (Quinlan and Hall, 2010). A set of three non-overlapping intervals encompassing a region that ranges from 204,479 to 3,926,269 bp in the chromosome 9 of the GDDH13.1.1 assembly was defined as loci of interest to mine the BBD QTL for candidate genes.

These intervals underlying the BBD QTL in our analysis comprised 17 markers (Supplementary Table S5; highlighted) and 219 predicted protein-coding gene structures, which were evaluated for prioritizing candidate genes (Supplementary Table S8). The list of gene models was carefully inspected giving priority to predicted genes that fitted in one or more of the following criteria: (i) related to cold response; (ii) functioning in dormancy and/or flowering time regulation; (iii) related to hormonal pathways, including absisic acid (ABA), GAs, and auxin-mediated signaling (Beauvieux et al., 2018); and (iv) having putative *cis*-acting elements in the promoter sequences thought to respond to chilling, such as those containing MYB and MYC motifs (Zhang et al., 2016).

In addition to relevant genes previously described in the literature at the top of the major QTL in LG9, like *MdoFLC* (MD09G1009100) and *MdoPRE1* (MD09G1049300) (Porto et al., 2015), we identified a predicted gene model (MD09G1003800, Chr09:335,088-338,411) that shares similarity to Arabidopsis *ICE1* (Supplementary Figure S3). The bHLH type transcription factor ICE1 binds to the promoter of the *CBF* genes to activate the *ICE1-CBF-COR* (COld Responsive genes) transcriptional cascade in plants, also known as *CBF* regulon (Chinnusamy et al., 2007; Thomashow, 2010; Ding et al., 2015; Park et al., 2015). The association of an apple *ICE1* (*MdoICE1*) to dormancy traits (CR, BBD, and flowering) agrees and complements the results observed when a peach *CBF* (*PpCBF1*) gene was constitutively expressed in an apple rootstock variety, resulting in altered expression of apple dormancy-related genes (*DAM* genes), early senescence in the autumn and delayed bud break in the spring (Wisniewski et al., 2015). Within this context, in order to investigate the *MdoICE1* participation in dormancy regulation, we determined its expression in different tissues and among a collection of individuals from the mapping population that showed stable early and late BBD across the years evaluated (Supplementary Figure S4). The data obtained demonstrated that *MdoICE1* is expressed in young/mature leaves; however, the transcriptional profiles did not reveal potential associations with the early neither the late bud break phenotypes. These results were somehow expected because activation of the *CBF* regulon relies on *ICE1* post-translational modifications triggered by cold stimulus (Ding et al., 2015). Taking into account that the transcriptional activity of the *ICE1* locus in itself cannot be associated with its biological function, we tested the CBF regulon transcriptional activity in response to cold stimulus using *in vitro* apple plants. The results gathered from this assay showed a slight but not significant induction of *MdoICE1*, followed by a significant sequential induction of *MdoCBFs* and *MdoDAM1*, a major candidate gene that has been associated with dormancy regulation in apple (Porto et al., 2016; Supplementary Figure S5). These data reinforce the findings reported by Wisniewski et al. (2015) and suggest an important role of the *MdoICE1*/*CBF* regulon in the control of bud dormancy in apple.

In the same region, we identified another gene (MD09G1004400, Chr09:368,274-371,719) characterized as mitochondrial phosphate transporter (MPT). The mRNA sequence for this gene showed high similarity with the *MPT* transcript EU072922, which was previously reported to be involved in accelerating bud dormancy release during chilling treatment in tree peony (*Paeonia suffruticosa*) (Huang et al., 2008; Zhang et al., 2016). The evidence for correlation of apple *ICE1* (*MdoICE1*) and MPT (*MdoMPT*) to traits associated to CR, BBD, and flowering reinforces a new perspective on the balance between different pathways in the control of dormancy release, integrating cold temperature perception, flowering, carbohydrate metabolism, and mitochondrial respiration. The relative positions of the main candidate genes within the BBD QTL on LG 9 are depicted in Supplementary Figure S6.

## Discussion

We present a genetic analysis of the BBD locus in a Full-Sib family derived from a controlled pollination F_1_ cross between contrasting genotypes for CR. The observed phenology for BBD suggests a strong genetic control of trait variation. In our study, broad sense heritability values were found high for BBD, 0.54 at BG and 0.73 at VC, reinforcing the appreciate contribution of underlying genes to the observed phenotypic variance. Albeit the heritability estimate was shown to be specific to the population and environment analyzed (de Souza et al., 1998), there are previous studies that have produced similar estimates for the heritability of the BBD trait in apple and peach (Fan et al., 2010; van Dyk et al., 2010; Celton et al., 2011). The similarity of values calculated from different progenies in different environments suggests a common genetic mechanism contributing for the phenotypic variance of traits associated with CR. Furthermore, despite the lower heritability and the lower explanation observed in BG, it is worth to note that sites with mild winters made it possible to exploit the genotype in such environment, favoring the observation of early BBD genotypes.

The constructed genetic linkage maps showed 17 LGs in accordance with Maliepaard et al. (1998) and corresponding to the haploid number of chromosomes in apple (Velasco et al., 2010). Using *whole-genome* sequencing of parental germplasm “M13/91” and “Fred Hough” and mapping short sequence reads to the reference, 182 new SNP markers were developed to be used in KASP assay for SNP genotyping. Using these markers for genotyping, we successfully increased the marker density to improve QTL mapping efficiency in detecting putative novel contributors to the BBD trait variability. The 784 grouped markers for “M13/91” and the 791 for “Fred Hough” revealed good marker saturation in all LGs. Linkage maps constructed using SSR or SNP markers from different apple (van Dyk et al., 2010; Celton et al., 2011; Antanaviciute et al., 2012; Zhang et al., 2012; Clark et al., 2014) and pear (Yamamoto et al., 2014; Chen et al., 2015; Gabay et al., 2017) populations showed similar coverage.

The linkage maps of both parents enabled an efficient detection of a major QTL affecting BBD. This QTL explained 24.4–53.30% of the variability at BG and 31–63% at VC, besides co-localizing with the genomic region associated with BBD and flowering time on LG9 beginning specified by van Dyk et al. (2010), Celton et al. (2011), Allard et al. (2016), and Urrestarazu et al. (2017). The identification of a common QTL in different segregating populations, climatic conditions, and years is an unusual finding. This result reinforces the importance of this region for the genetic control of BBD in apple trees and incentive investigations of the underlying genes contributing effects to the trait variance. Although this region explains more than 50% of the phenotypic variation observed for BBD, other important regions have also been reported on LG7, LG10, and LG12, as well as QTL with minor effects on LG8 and LG15 (Allard et al., 2016). Among the progress toward the search for candidate genes putatively associated with dormancy traits, the interval of this QTL was initially defined as the first 4.04 Mb (Celton et al., 2011) and subsequently narrowed to 1.8(Allard et al., 2016), 1.7 (Trainin et al., 2016), and 0.36 Mb (Urrestarazu et al., 2017). The segments indicated in these contributions overlap with the physical interval defined in our study (3.28 Mb), which was defined based on the stability of the QTL across years and sites.

The genetic control of dormancy in Rosaceae is a complex process and the identification of major genes controlling this trait are challenging. In the beginning of apple chromosome 9 (position 655,000), a sequence with similarity to *FLC* (MD09G1009100; *MdoFLC*) was already described as a strong candidate to have a functional role during flowering (Porto et al., 2015; Allard et al., 2016; Urrestarazu et al., 2017). *MdoFLC* was shown to be seasonally expressed during dormancy (Porto et al., 2015), and recently, this gene was shown to be differentially expressed when comparing ecodormant buds to bud break and fruit set stages (Kumar et al., 2017), suggesting a putative role during endo- to ecodormancy transition. In Arabidopsis, the *FLC* gene is the main repressor of flowering under unfavorable conditions (reviewed in Amasino and Michaels, 2010).

Besides *MdoFLC*, genes from other functional classes were identified inside the confidence intervals of the LG9 BBD QTL such as NAC-domain protein (MD09G1006400), putative WRKY transcription factor (MD09G1008800), and chromatin-remodeling complex (MD09G1011500; MD09G1011600) (Celton et al., 2011; Allard et al., 2016; Trainin et al., 2016; Urrestarazu et al., 2017). Although intense efforts have been made to trace candidate genes that can play roles in the genetic control of dormancy induction and release, a biological integration of these findings is still missing.

Within this context, we carefully inspected the physical interval of the LG9 BBD QTL for new candidate genes that could be primarily associated with cold response, dormancy or ABA-mediated signaling. We identified a gene model located close to the highest significant LOD scores (Supplementary Tables S5, S8) that shows similarity to an Arabidopsis gene encoding the bHLH transcription factor ICE1 (Supplementary Figure S3). Apples and pears diverge in bud dormancy regulation from other Rosaceae species because instead of being triggered by photoperiodic changes, the main regulator of this process is exposure to cold temperatures (Heide and Prestrud, 2005). Therefore, the identification of *MdoICE1* inside the apple BBD QTL gives us the opportunity to add new evidence to the elucidation of dormancy control: a candidate gene associated with cold perception and flowering regulation. Apart from being located close to the highest significant LOD scores across the years evaluated in two different sites (Supplementary Tables S5, S8), it is well stablished in the literature that upon cold stimulus, ICE1 and its targets *CBF* transcription factors activate downstream cold-responsive gene targets, including COLD-REGULATED (COR) genes also known as the CBF regulon (Gilmour et al., 1998; Chinnusamy et al., 2003, 2007; Thomashow, 2010; Kim et al., 2015; Park et al., 2015; Zhan et al., 2015). In addition, ICE1 can also directly integrates cold signals into *FLC*-mediated flowering pathways, and ICE1 activity is post-translationally modulated by OST1, a key component in ABA signaling (Ding et al., 2015; Lee et al., 2015). Under floral promoting conditions, ICE1 binding to *FLC* and *CBF* promoters is inhibited and leads to flowering induction (Lee et al., 2015). In apple, MdCIbHLH1 (MD14G1148600), an ICE-like protein has the ability to promote chilling tolerance in transgenic plants; however, this gene has not been assigned to QTL positions for dormancy traits (Feng et al., 2012). This collection of evidences suggests *MdoICE1* as an appealing candidate gene to mediate cold and ABA responsiveness by activating the CBF regulon during the bud dormancy process in apple and that a balance between different pathways are acting to mediate adaptive responses to cold perception.

Interestingly, CBFs were shown to modulate apple dormancy by altering the expression of genes responsible for the molecular control of dormancy progression such as the *DAM* genes (Wisniewski et al., 2015; Artlip et al., 2016). Similarly, the pear CBF2 was shown to interact with the promoter of the *DAM* ortholog *PpMADS13-1* (Saito et al., 2015), a trend that was already suggested for *DAM* genes of other species (Horvath et al., 2010; Sasaki et al., 2011; Yamane et al., 2011; Porto et al., 2016). The context of these evidences together with the data gathered in this work can also contribute to the understanding of the genetics behind dormancy traits. As part of the CBF regulon, *MdoDAM* genes function may be directly affected by the genetic variance of their upstream regulators, therefore establishing a potential epistatic effect. This might be one of the explanations why QTL associated with *MdoDAM* chromosomal positions in apple were not found in bi-parental progenies (this study; van Dyk et al., 2010; Celton et al., 2011) and even using a large and powerful GWAS study (Urrestarazu et al., 2017). Most *MdoDAM* genes were found at LG8 in a region highly syntenic to the peach chromosome 1, where the *PpDAM* genes are located (Porto et al., 2016). Hence, if conserved genetic mechanisms involving *DAM* genes in peach were to be found in apple, they are expected to map to apple LG8. Allard et al. (2016) exploring a pedigree-based analysis in multi-parental populations were able to detect QTLs for bud phenology traits associated with *MdoDAM* chromosomal positions on LG8 and LG15 in two of the three years evaluated; however, they were considered of minor contribution.

In addition, we identified in the same QTL interval a gene model showing high similarity to *MPT* (*MdoMPT*), a gene involved in accelerating bud dormancy release during chilling treatment in tree peony (Huang et al., 2008; Zhang et al., 2016). Cold-responsive genes are able to encode a diverse array of proteins involved in respiration and metabolism of carbohydrates (Chinnusamy et al., 2003). Noteworthy, the carbohydrate metabolism appears essential in the transition from dormancy to active bud growth in response to cold. The bud capacity to burst was suggested to be tightly linked to its carbohydrates supply due to increasing carbohydrate uptake in the bud after dormancy release, with an increase in the expression and activity of membrane transporters (Beauvieux et al., 2018). Remarkably, the *MPT* expression regulation in dormancy release is thought to be essential to promote respiratory rate and energy metabolism, which is a process that requires delivering of inorganic phosphate to the mitochondrial ATP synthase complex (Beauvieux et al., 2018). The identification of *MdoMPT* in the LG9 QTL adds carbohydrate metabolism and mitochondrial respiration pathways into the likely players of dormancy in apple.

**FIGURE 3:**
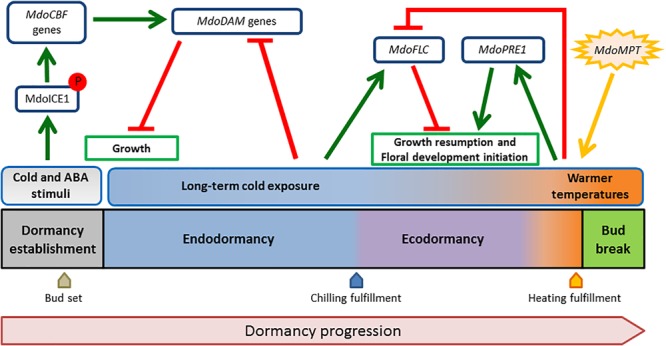
The proposed hypothetical model for the apple bud dormancy process. The model depicts schematically the dormancy stages from dormancy establishment to bud break. Environmental stimuli are presented above the dormancy stages. Potential regulatory roles of candidate genes are shown in dark blue boxes. Green arrows indicate putative transcriptional activation while T end red bars represent repression activity. The orange star-shaped form represents source of energy. Post-translational modification of MdoICE1 (phosphorylation and stabilization) upon stimuli is indicated with P embedded in red.

Taking advantage of the known functional pathways of ICE1, CBF, and FLC in Arabidopsis, we suggest a hypothetical model for the apple bud dormancy process based on the findings of the LG9 BBD QTL (Figure 3). Early cold waves and ABA stimuli activate the MdoCBF regulon through MdoICE1. *MdoDAM* genes would be activated leading to dormancy establishment. Medium- to long-term cold exposure have a role during endo- to ecodormancy transition by activating *MdoFLC* through the same pathway, but in a CR-dependent manner. The balance of both *MdoDAM* and *MdoFLC* levels would guarantee that dormancy release and bud break only occur during optimal growth promoting conditions after heat requirement fulfillment. However, how the same pathway would induce these genes in different time points is yet to be discovered.

Complementarily, the examination of the BBD QTL on the M13/91 progenitor revealed two remarkable peak regions dividing the QTL depending on the threshold adopted and year evaluated (see Supplementary Table S5): the first at the extremity (marker TC_465054_Lg9) and the second one starting around 9–11 cM (markers drm_qtl_snprnt113 and drm_qtl_snprnt137). The marker drm_qtl_snprnt137 lies within the MD09G1049300 gene model, which was annotated as an ortholog of *PRE1*. The LOD distribution drops to non-significant values immediately after this marker’s position, except in the atypical winter of 2017 (see Figure 1 and Supplementary Tables S1, S2, S5). This finding suggests a relevant role of *MdoPRE1* locus for the phenology of dormancy-associated traits (bud break and flowering). In agreement to the genetic analysis, differential expression of *MdoPRE1* during bud break was observed in apple (Porto et al., 2015; Kumar et al., 2017), reinforcing a potential role in growth resumption during bud dormancy release. This set of observations also accounts for a potential additive effect between the loci located at the borders of the LG9 BBD QTL, emphasizing the complex genetic control of this trait. The additive effect hypothesis is also consistent because time of bud break and flowering are indirect measures of CR, and genetic analysis approaches based on phenotyping of these traits failed to identify loci responsible for the maintenance of the dormancy process (growth repressors) in apple trees (Celton et al., 2011; Allard et al., 2016; Trainin et al., 2016; Urrestarazu et al., 2017).

In the present study, the use of phenological data collected during 7 years in two locations with different climatic conditions allowed the dissection of different loci contributions to BBD. The results obtained allowed us to revisit the genetic characterization of the corresponding BBD QTL chromosomal segment, reinforcing the strong genetic effects over dormancy-associated traits. The proximity of candidate genes associated with chilling perception, ABA signaling, and flowering (*MdoICE1, MdoFLC*, and *MdoPRE1*) from the most explanatory markers suggests potential complementary roles of these genes functions in dormancy establishment and release. Several models for bud dormancy control have been proposed (Horvath, 2009; Campoy et al., 2011; Rinne et al., 2011; van der Schoot and Rinne, 2011). However, they are based on species in which photoperiod plays a major role in dormancy induction. These models do not address the peculiarities of this process in pipfruits, such as apples and pears. Therefore, a tentative summarizing model is proposed in Figure 3. The hypothetical model postulates that: *MdoICE* is important for cold perception to start the signaling involved in dormancy establishment and adaptive responses; the *MdoDAM* genes are important to induce bud set and to repress growth resumption until CR fulfillment; *MdoFLC* is important to inhibit growth after the transition from endo- to ecodormancy; *MdoPRE1* is important for bud break, possibly by mediating GA promotion of vegetative growth; and *MdoMPT* represents the energy source necessary for growth as being associated with carbohydrate metabolism. In the breeding context, the interpretation of the results presented in this work may help the development of strategies that could support the generation of new cultivars better adapted to each regional cultivation scenario, either by biotechnological or by conventional practices.

## Author Contributions

YEM was involved in the phenotypic analysis and KASP genotyping, as well as drafting the manuscript. CT was involved in phenotypic analysis and SNP genotyping using RosBREED markers. ABCC assisted in the management of the orchards, phenotypic and statistical analysis. DDP worked in the KASP markers development. DDP, VSF, OBdS-J, RCT, PG, MMCC and TS realized the *in silico* analysis of the candidate genes. SAdA, OBdS-J, RCT, MMCC, GJP and PG performed the *de novo* SNP discovery. VB and AMC performed the gene expression analysis. MVK and FD developed the F1 population. PRDO, CAD and LFR conceived and supervised the work, edited and finalized the manuscript.

## Conflict of Interest Statement

The authors declare that the research was conducted in the absence of any commercial or financial relationships that could be construed as a potential conflict of interest.

## Footnotes

1 https://iris.angers.inra.fr/gddh13/

2 http://genomewiki.ucsc.edu/index.php/Same_species_lift_over_construction#Same_Species_Lift_Over
