# Supplementary material for: Spring Is Coming: Genetic Analyses of the Bud Break Date Locus Reveal Candidate Genes From the Cold Perception Pathway to Dormancy Release in Apple (Malus × domestica Borkh.)
Source: Front Plant Sci. 2019 Mar 7;10:33. doi: 10.3389/fpls.2019.00033 (PMC6423911; doi:10.3389/fpls.2019.00033)
Supplement: Supplementary file 1 [file Presentation_1.zip › Supplementary Materials/Supplementary Figure 3.PDF]

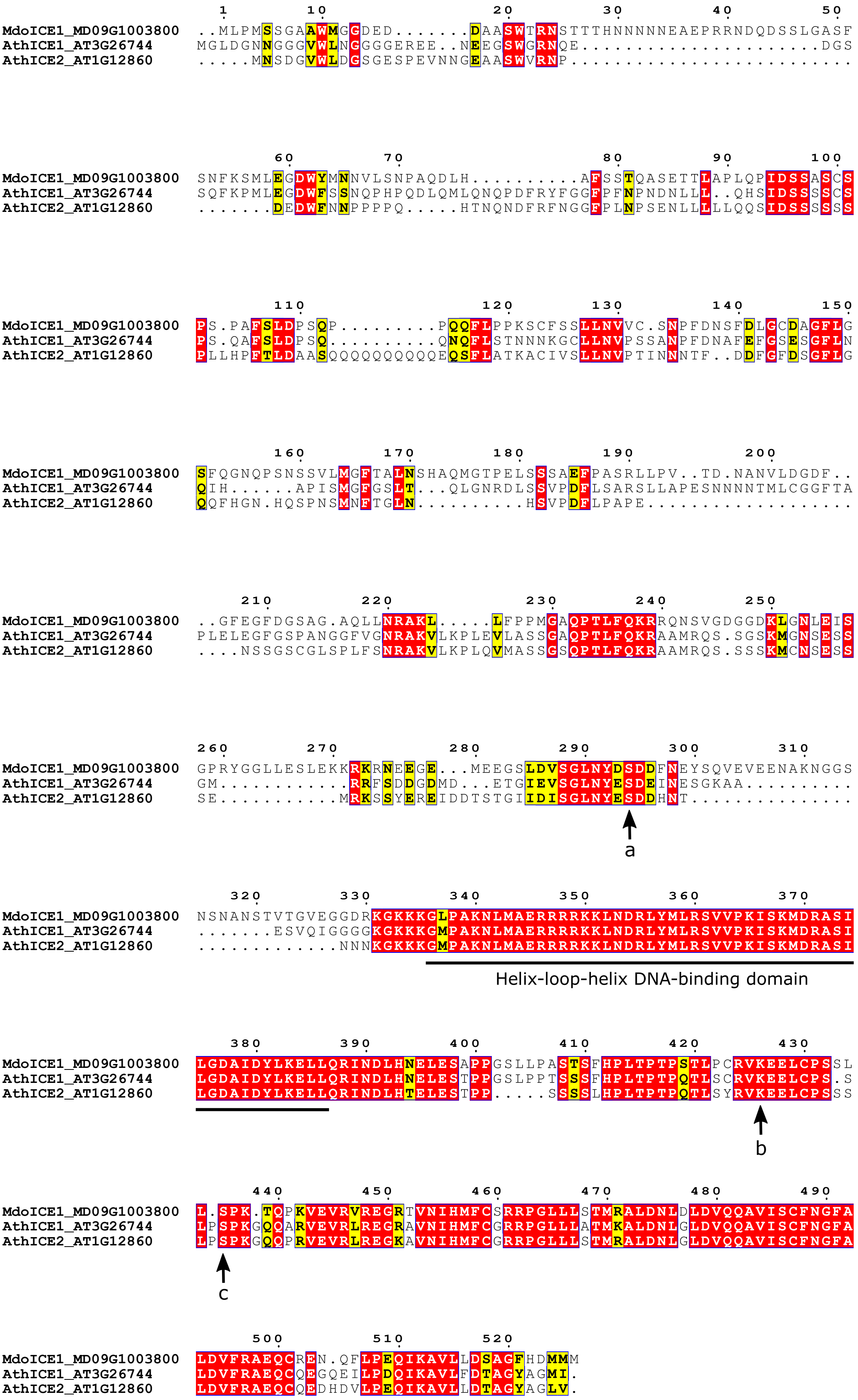
MdoICE1_MD09G1003800 ..MLPMSSGAAWMGGDED.......DAASWTRNSTTTHNNNNNEAEPRRNDQDSSLGASF
AthICE1_AT3G26744 MGLDGNNGGGVWLNGGGGEREE..NEEGSWGRNQE......................DGS
AthICE2_AT1G12860 .....MNSDGVWLDGSGESPEVNNGEAASWVRNP.........................
MdoICE1_MD09G1003800 SNFKSMLEGDWYMNNVLSNPAQDLH..........AFSSTQASETTLAPLQPIDSSASCS
AthICE1_AT3G26744 SQFKPMLEGDWFSSNQPHPQDLQMLQNQPDFRYFGGFPFNPNDNLLL..QHSIDSSSSCS
AthICE2_AT1G12860 .......DEDWFNNPPPPQ.....HTNQNDFRFNGGFPLNPSENLLLLLQQSIDSSSSSS
MdoICE1_MD09G1003800 PS.PAFSLDPSQP.........PQQFLPPKSCFSSLLNVVC.SNPFDNSFDLGCDAGFLG
AthICE1_AT3G26744 PS.QAFSLDPSQ..........QNQFLSTNNNKGCLLNVPSSANPFDNAFEFGSESGFLN
AthICE2_AT1G12860 PLLHPFTLDAASQQQQQQQQQQEQSFLATKACIVSLLNVPTINNNTF..DDFGFDSGFLG
MdoICE1_MD09G1003800 SFQGNQPSNSSVLMGFTALNSHAQMGTPELSSSAEFPASRLLPV..TD.NANVLDGDF..
AthICE1_AT3G26744 QIH......APISMGFGSLT...QLGNRDLSSVPDFLSARSLLAPESNNNNTMLCGGFTA
AthICE2_AT1G12860 QQFHGN.HQSPNSMNFTGLN..........HSVPDFLPAPE...................
MdoICE1_MD09G1003800 ..GFEGFDGSAG.AQLLNRAKL.....LFPPMGAQPTLFQKRRQNSVGDGGDKLGNLEIS
AthICE1_AT3G26744 PLELEGFGSPANGGFVGNRAKVLKPLEVLASSGAQPTLFQKRAAMRQS.SGSKMGNSESS
AthICE2_AT1G12860 ....NSSGSCGLSPLFSNRAKVLKPLQVMASSGSQPTLFQKRAAMRQS.SSSKMCNSESS
MdoICE1_MD09G1003800 GPRYGGLLESLEKKRKRNEEGE...MEEGSLDVSGLNYDSDDFNEYSQVEVEENAKNGGS
AthICE1_AT3G26744 GM............RRFSDDGDMD...ETGIEVSGLNYESDEINESGKAA..........
AthICE2_AT1G12860 SE...........MRKSSYEREIDDTSTGIIDISGLNYESDDHNT...............
a
MdoICE1_MD09G1003800 NSNANSTVTGVEGGDRKGKKKGLPAKNLMAERRRRKKLNDRLYMLRSVVPKISKMDRASI
AthICE1_AT3G26744 .......ESVQIGGGGKGKKKGMPAKNLMAERRRRKKLNDRLYMLRSVVPKISKMDRASI
AthICE2_AT1G12860 .............NNNKGKKKGMPAKNLMAERRRRKKLNDRLYMLRSVVPKISKMDRASI
Helix-loop-helix DNA-binding domain
MdoICE1_MD09G1003800 LGDAIDYLKELLQRINDLHNELESAPPGSLLPASTSFHPLTPTPSTLPCRVKEELCPSSL
AthICE1_AT3G26744 LGDAIDYLKELLQRINDLHNELESTPPGSLPPTSSSFHPLTPTPQTLSCRVKEELCPS.S
AthICE2_AT1G12860 LGDAIDYLKELLQRINDLHTELESTPP.....SSSLHPLTPTPQTLSYRVKEELCPSSS
b
MdoICE1_MD09G1003800 L.SPK.TQPKVEVRVREGRTVNIHMFCSRRPGLLLSTMRALDNLDLDVQQAVISCFNGFA
AthICE1_AT3G26744 LPSPKGQQARVEVRLREGRAVNIHMFCGRRPGLLLATMKALDNLGLDVQQAVISCFNGFA
AthICE2_AT1G12860 LPSPKGQQPRVEVRLREGKAVNIHMFCGRRPGLLLSTMRALDNLGLDVQQAVISCFNGFA
c
MdoICE1_MD09G1003800 LDVFRAEQCREN.QFLPEQIKAVLLDSAGFHDMMM
AthICE1_AT3G26744 LDVFRAEQCQEGQEILPDQIKAVLFDTAGYAGMI.
AthICE2_AT1G12860 LDVFRAEQCQEDHDVLPEQIKAVLLDTAGYAGLV.
