# Supplementary material for: Spring Is Coming: Genetic Analyses of the Bud Break Date Locus Reveal Candidate Genes From the Cold Perception Pathway to Dormancy Release in Apple (Malus × domestica Borkh.)
Source: Front Plant Sci. 2019 Mar 7;10:33. doi: 10.3389/fpls.2019.00033 (PMC6423911; doi:10.3389/fpls.2019.00033)
Supplement: Supplementary file 1 [file Presentation_1.zip › Supplementary Materials/Supplementary Figures and Tables Captions.pdf]

**Spring is coming: genetic analyses of the bud break date locus reveal candidate genes from the cold perception pathway to dormancy release in apple (*Malus* × *domestica* Borkh.)**

**Authors**

Yohanna Evelyn Miotto[1,8*], Carolina Tessele[1*], Ana Beatriz Czermainski[2], Diogo Denardi Porto[2,9], Vítor da Silveira Falavigna[2,3,10], Tiago Sartor[2,3], Amanda Malvessi Cattani[2,3], Carla Andrea Delatorre[1], Sérgio Amorim de Alencar[4], Orzenil da Silva-Junior[5], Roberto Coiti Togawa[5], Marcos Mota do Carmo Costa[5], Georgios Joannis Pappas Jr[6], Priscila Grynberg[5], Paulo Ricardo Dias de Oliveira[2], Marcus Vinícius Kvitschal[7], Frederico Denardi[7], Vanessa Buffon[2], Luís Fernando Revers[2,3,§]

[1]Department of Crop Science, Agronomy School, Federal University of Rio Grande do Sul, Porto Alegre, RS, 91501-970, Brazil

[2]Embrapa Uva e Vinho, Bento Gonçalves, RS, 95701-008, Brazil

[3]Graduate Program in Cell and Molecular Biology, Centro de Biotecnologia, Federal University of Rio Grande do Sul, Porto Alegre, RS, 91501-970, Brazil

[4]Programa de Pós-Graduação em Ciências Genômicas e Biotecnologia, Universidade Católica de Brasília, Brasília-DF, Brazil, 70770-900, Brazil

[5]Embrapa Recursos Genéticos e Biotecnologia, Brasília, DF - 70770-917, Brazil

[6]Department of Cell Biology, Universidade de Brasília, Brasília, DF 70910-900, Brazil

[7]Empresa de Pesquisa Agropecuária e Extensão Rural de Santa Catarina – Epagri – Estação Experimental de Caçador, Caçador, SC, 89500-000, Brazil.

[8]Present Address: Programa de Pós-Graduação em Genética e Biologia Molecular, Federal University of Rio Grande do Sul, Porto Alegre, RS, 91501-970, Brazil

[9]Present Address: Embrapa Semiárido, Petrolina, PE, 56302-970, Brazil

[10]Present address: AGAP, Univ. Montpellier, CIRAD, INRA, Montpellier SupAgro, Montpellier, France

[§]Corresponding author

**Dr. Luís Fernando Revers**

Laboratory of Plant Molecular Genetics, Embrapa Uva e Vinho

Rua Livramento, 515, P.O. Box 130, CEP: 95701-008, Bento Gonçalves, RS, Brazil

**Tel:** +55 54 3455 8039
**Fax:** +55 54 3455 8127
**E-mail:** luis.revers@embrapa.br

*These authors contributed equally to this work.

# 1 Supplementary Data

## 1.1 Supplementary Figures

**Supplementary Figure 1**. Parental genetic linkage maps of 'Fred Hough' and 'M13/91'. Parental maps were aligned using common markers. Numbering of LGs are according to Maliepaard et al. (1998).

**Supplementary Figures 2.** Association of BBD markers with the phenotypic variation for *Malus* x *domestica* cultivars 'M13/91' and 'Fred Hough' grown in Bento Gonçalves (A) and in Vacaria (B). Fifteen selected markers from the BBD QTL interval are presented along with their respective genotype vs. BBD phenotype. For each year, the BBD trait phenotype was subdivided into quartiles. Early represents plants within the earlier (first) quartile; Middle represents the plants within the second and third quartile; and Late represent the plants within the fourth quartile.

**Supplementary Figure 3.** Sequence alignment of *M. domestica* ICE1 (MdoICE1) and *A. thaliana* ICE1 and ICE2 (AthICE1 and AthICE2) proteins. Identical amino acids are highlighted by white bold letters inside red boxes, while similar or chemically equivalent amino acids are highlighted by black bold letters inside yellow boxes. Amino acid residues known to be subjected to post-translational modifications in *A. thaliana* are indicated by arrows as follows: a) serine residue phosphorylated by OST1; b) lysine residue needed for SIZ1-mediated sumoylation; c) serine residue necessary for HOS1-mediated ubiquitination of AthICE1.

**Supplementary Figure 4.** Gene expression analysis of *MdoICE1*. **A**) Gene expression of *MdoIce1* in 14 different organs and tissues of cv. Gala Brookfield. The relative expression of *MdoIce1* was evaluated in different growth stages following

the Fleckinger’s apple phenological classification (EPPO, 1984). (A) closed terminal buds; (B) buds with green tip (C) bud bursting with emerging leaves; (E2 YL) young leaves; (E2 FB) flower buds (I leaf) leaves at I stage; (I WSF) whole set-fruits with approximately 10mm diameter; (J ML) mature leaves and unripe fruits with approximately 40 mm diameter which were divided into pulp, seed, and skin; (M) mature fruits with approximately 70 mm diameter partitioned into pulp, seed and skin. The expression data was calculated in relation to A stage. Statistical analysis was performed by One-way ANOVA followed by Tukey test with GraphPad Prism 6 software ($p < 0.05$). **B**) Division of the BBD period of each year in three sub periods based on the first and third quartiles (Q1 and Q3) estimated for the whole population in Bento Gonçalves, RS, resulting in the classification of the plants in early (BBD < Q1), intermediate (Q1 < BBD < Q3) and late (BBD > Q3) bud breaking. **C**) Gene expression of *MdoIce1* in early and late bud break individuals from the segregating population. The relative expression of *MdoIce1* were evaluated in 15 early (EB) and 7 late (LB) bud break individuals from the cross between ‘M13/91’X‘Fred Hough’. Statistical analysis was performed by One-way ANOVA followed by Tukey test with GraphPad Prism 6 software ($p < 0.05$). Sampling and RT-qPCR for **A** and **C** were performed as described in Perini et al (2014) and Falavigna et al. (2014) respectively. Primers are listed in Supplementary Table 9.

**Supplementary Figure 5.** Treatment of cold exposure of apple plantlets *in vitro*. Relative expression of *MdoIce1*, *MdoCBF1*, *MdoCBF2*, *MdoCBF3* and *MdoDAM1* were evaluated in four weeks old plantlets of ‘Gala Brookfield’ treated by 0, 3, 6, 24 and 48h of chilling (4°C). Plantlets were sampled in triplicates. Each triplicate consisted of three plantlets per sampling time. Statistical analysis comparison was performed to each gene individually in relation to time 0h. One-way ANOVA followed by Dunnet test with GraphPad Prism 6 software were used to perform statistical analysis (*$0.01 < p < 0.05$, **$0.001 < p$ <0.01***, $p < 0.001$****). RT-qPCR analysis was performed as described by Falavigna et al. (2014). Primers are listed in Supplementary Table 9.

**Supplementary Figure 6.** Distribution of LOD values and position of the candidate genes *MdoICE1, MdoMPT, MdoFLC* and *MdoPRE1* along the chromosome 9 for the “M13/91” BBD QTL at Bento Gonçalves (A) and in Vacaria (B). The actual position

of each mark was determined based on the reference genome published by Daccord *et al*. (2017). LOD values associated with each mark were calculated by Interval Mapping using MapQTL® v6 (Kyazma®, Netherlands) as described in Material and Methods using the physical distances instead of the ones calculated based on the frequencies of recombination. This analysis was performed to plot the LOD distribution avoiding inversions relative to the reference genome when linkage groups were generated with JoinMap® v4 (Kyazma®, Netherlands). LOD thresholds are listed in Supplementary Table 10. The scale represents distances in Mb.

## 1.2 Supplementary Tables

**Supplementary Table 1.** Temperature and rainfall records of Bento Gonçalves and Vacaria, from 2011 until 2017.

**Supplementary Table 2.** Accumulated chill units according to North Carolina model (Shaltout and Unrath, 1983), modified by Ebert et al. (1986), for Bento Gonçalves (BG) and Vacaria (VC), RS, Brazil.

**Supplementary Table 3.** KASP marker sequences of the 182 new single nucleotide polymorphisms (SNPs) within the QTL interval on LG9. Polymorphisms are indicated in brackets.

**Supplementary Table 4.** Basic characteristics of the parental genetic linkage groups for ‘M13/91’ and ‘Fred Hough’. cM: centiMorgan units.

**Supplementary Table 5.** Quantitative trait loci detected for bud break (BBD) date on linkage group 9 (LG9) using restrict multiple QTL mapping in the ‘M13/91’ x ‘Fred Hough’ segregating population in different years in Bento Gonçalves and Vacaria. Maximum LOD scores are presented with considered linkage group (LG) and genome-wide (GW) LOD thresholds shown below year indication along with percentage of the variance explained by the QTL (% exp.). Average indicate the LOD and percentage of the variance explained by the QTL taking into account the mean values of the phenotypic data across the years. Markers highlighted in green indicate the loci of interest to mine the BBD QTL for candidate genes.

**Supplementary Table 6.** Chi-square independency test between phenotypic and genotypic association of markers for BBD. Markers, corresponding chi-square values, and p values are presented by planting site (BG and VC) and year of growing cycle.

**Supplementary Table 7.** Wilcoxon-rank test to test if genes that belong to one category do not differ in their ranks from genes that do not belong to the category. The average LOD for the trait by marker in the MapQTL6 analysis using the phenotypic traits assessment from site locations BG and VC was used as a measure of choice to rank the genes (see Supplementary Table 5).

**Supplementary Table 8.** List of gene models of the BBD QTL interval inspected for prioritizing candidate genes. Gene models are listed with corresponding function description, localization and links to the apple genome and epigenome database. The main candidate genes are marked in yellow.

**Supplementary Table 9.** List of primers used for RT-qPCR analysis.

**Supplementary Table 10**. LOD thresholds calculated for the BBD QTL described in Supplementary Figure 6 for the parent "M13/91". LOD threshold values calculated for each year are indicated per linkage group (LG) and genome wide (GW) respectively (LG/GW). nd = not determined.
