# Supplementary figures and images for: Spring Is Coming: Genetic Analyses of the Bud Break Date Locus Reveal Candidate Genes From the Cold Perception Pathway to Dormancy Release in Apple (Malus × domestica Borkh.)
Source: Front Plant Sci. 2019 Mar 7;10:33. doi: 10.3389/fpls.2019.00033 (PMC6423911; doi:10.3389/fpls.2019.00033)

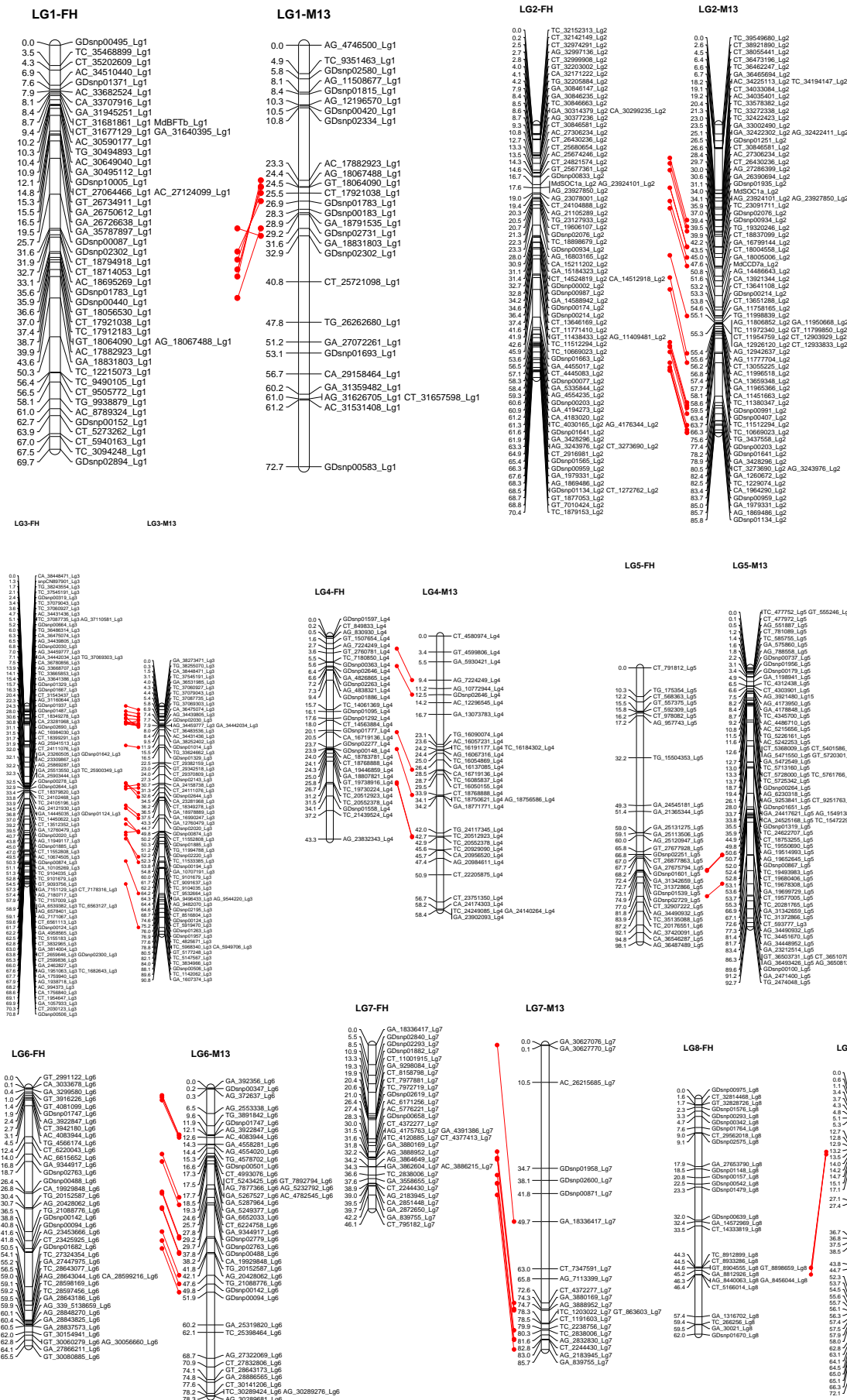

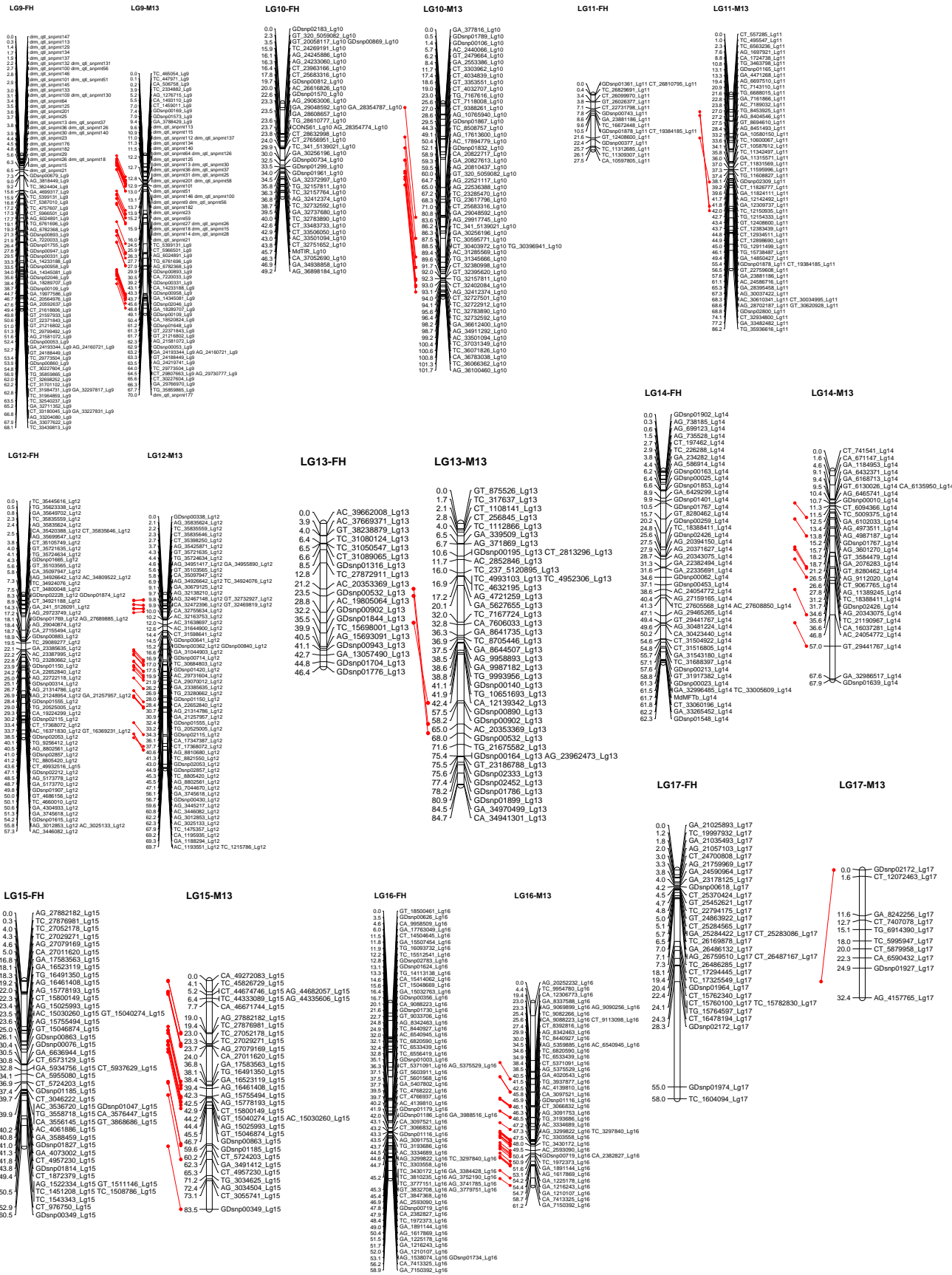

Supplement: Supplementary file 1 [file Presentation_1.zip › Supplementary Materials/Supplementary Figure 1.PDF]

(A) Bento Gonçalves - BG

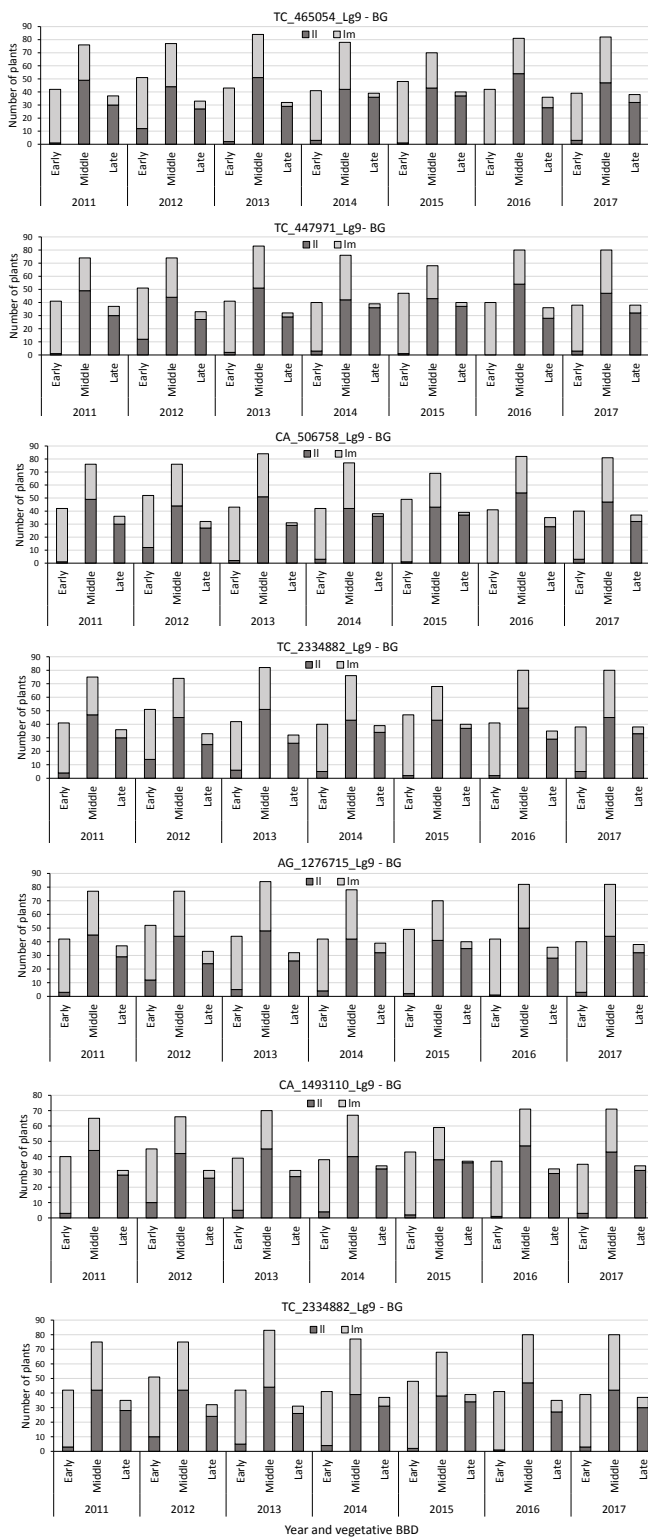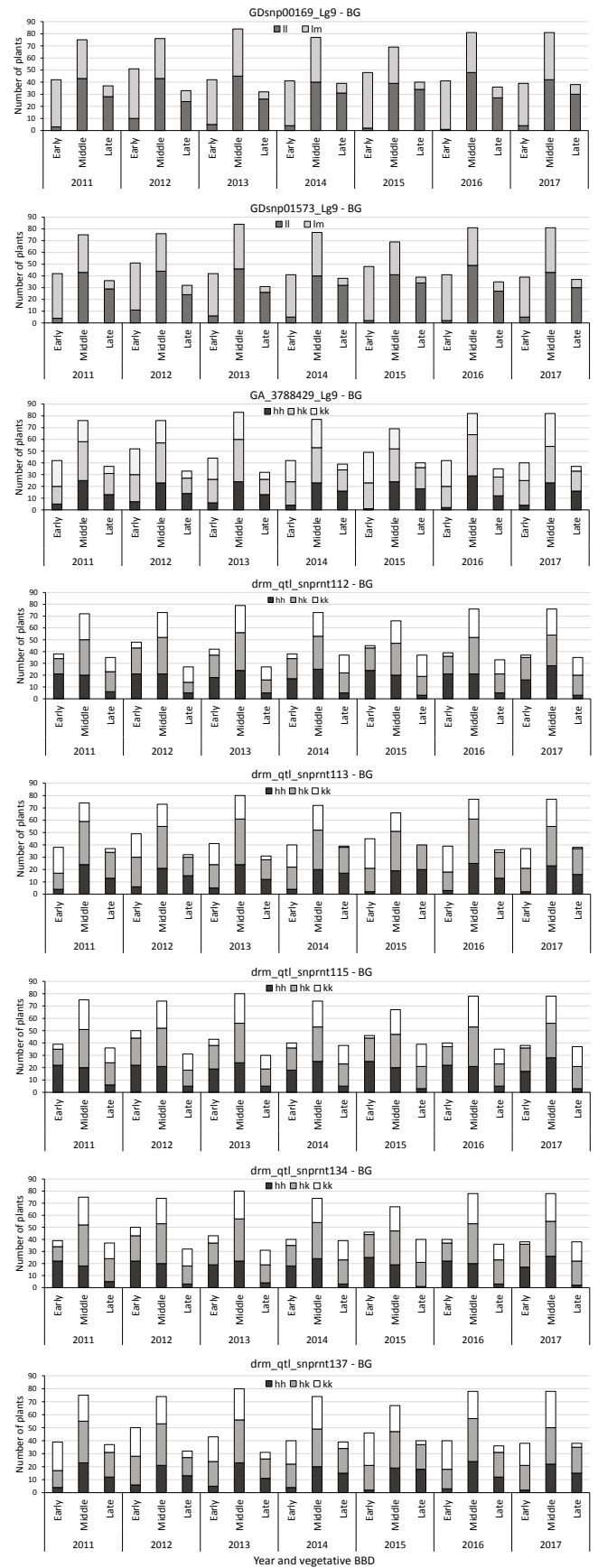

(B) Vacaria - VC

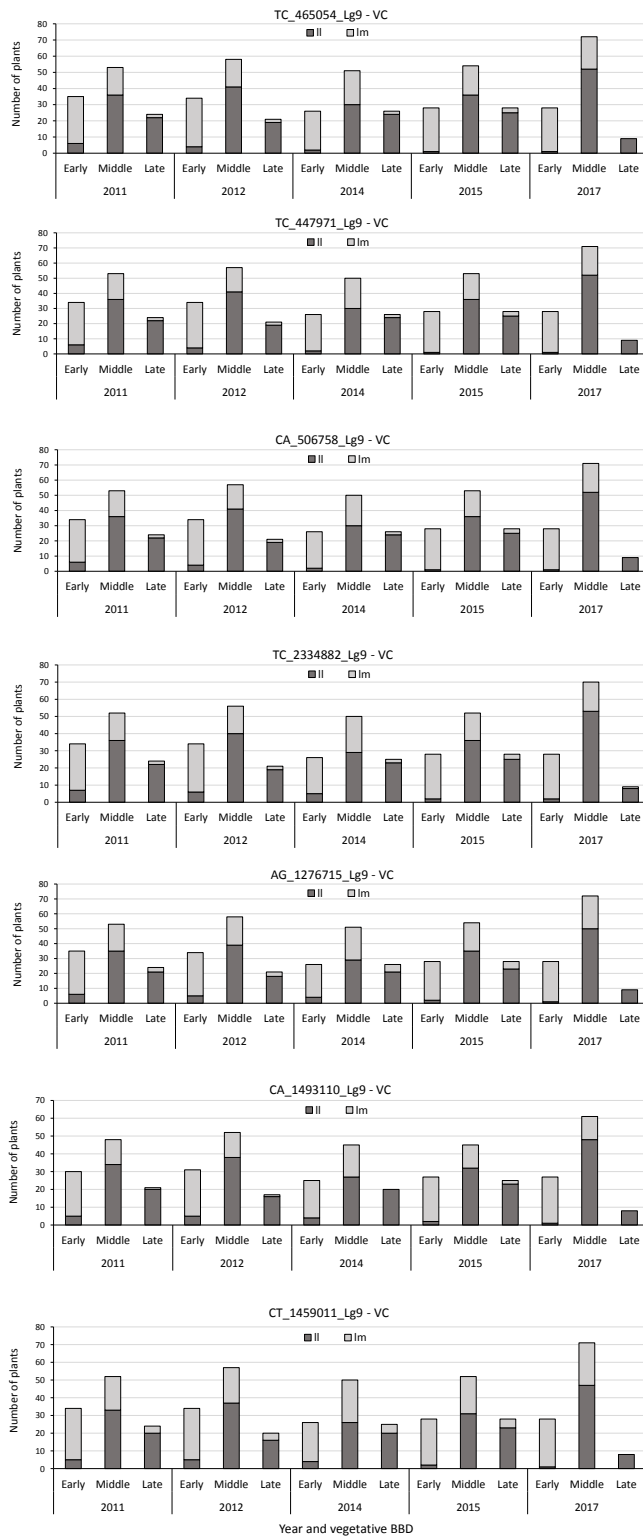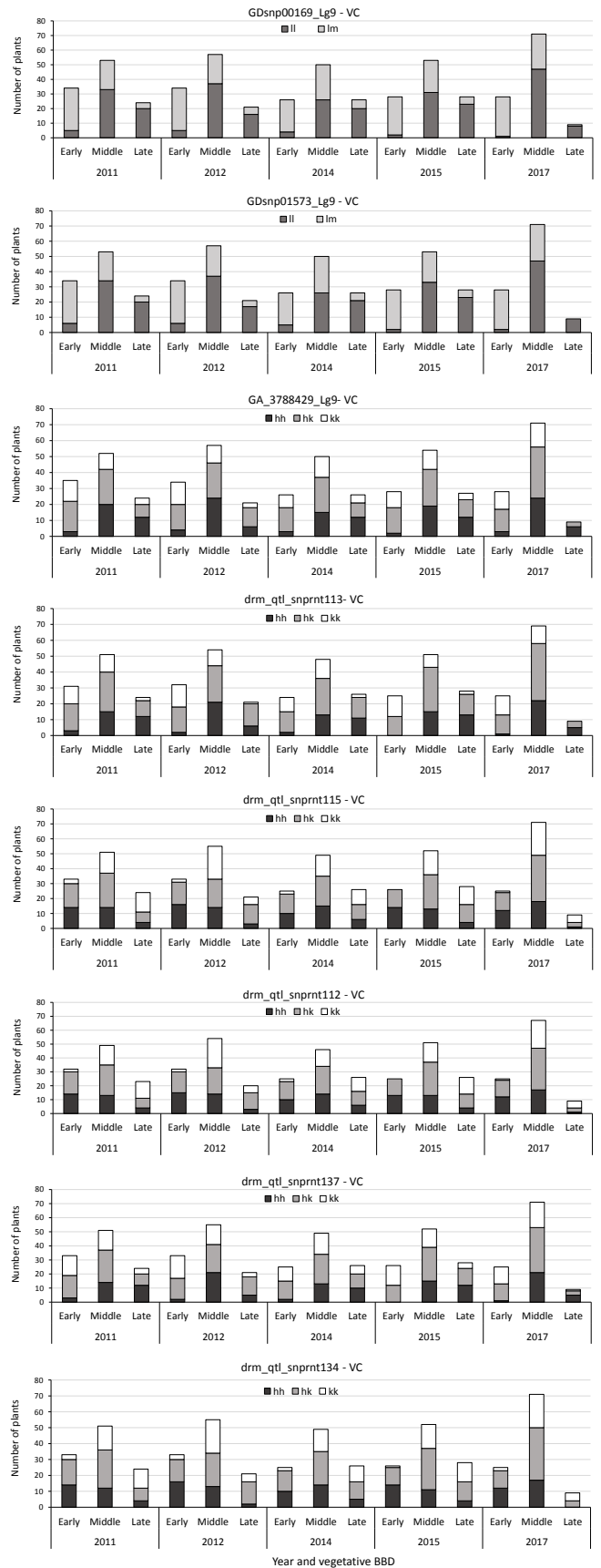

Supplement: Supplementary file 1 [file Presentation_1.zip › Supplementary Materials/Supplementary Figure 2.PDF]

# Supplementary Figure 4

A

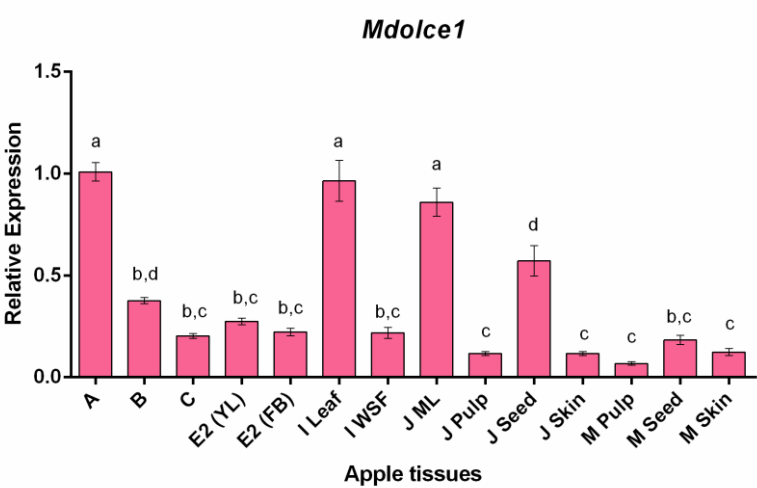

B

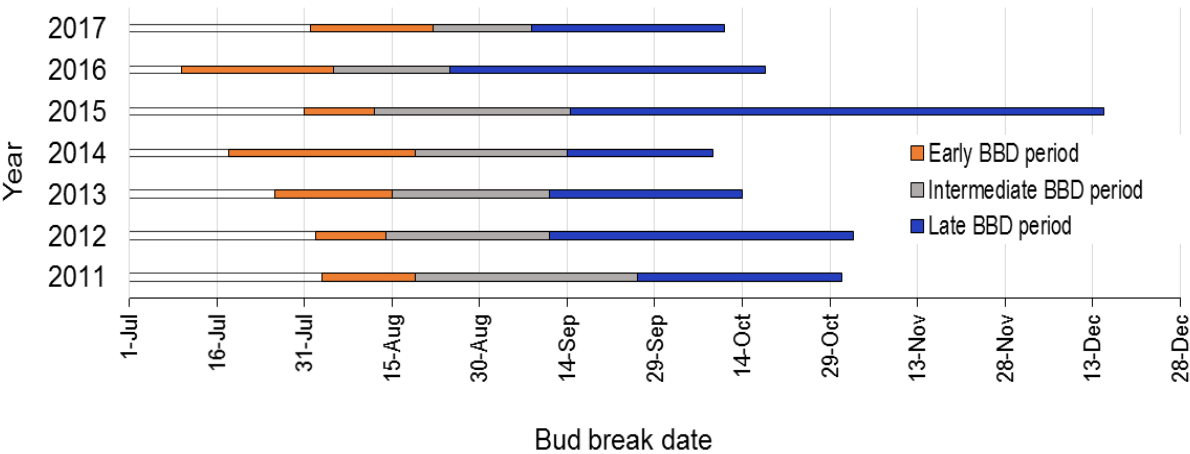

C

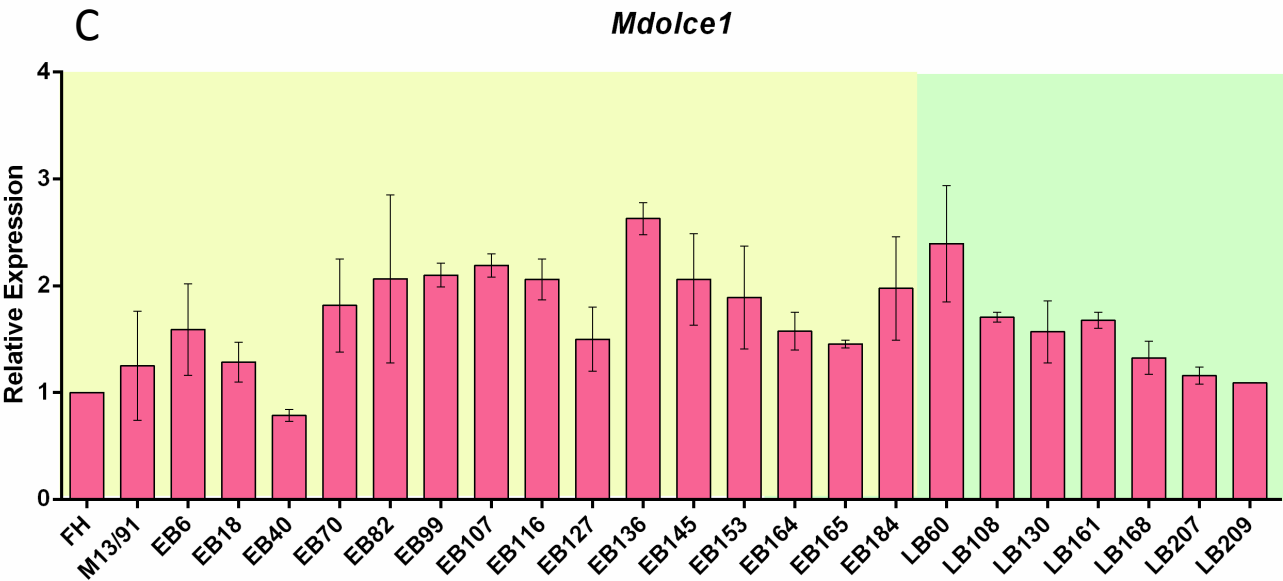

Supplement: Supplementary file 1 [file Presentation_1.zip › Supplementary Materials/Supplementary Figure 4.pdf]

Supplementary Figure 5

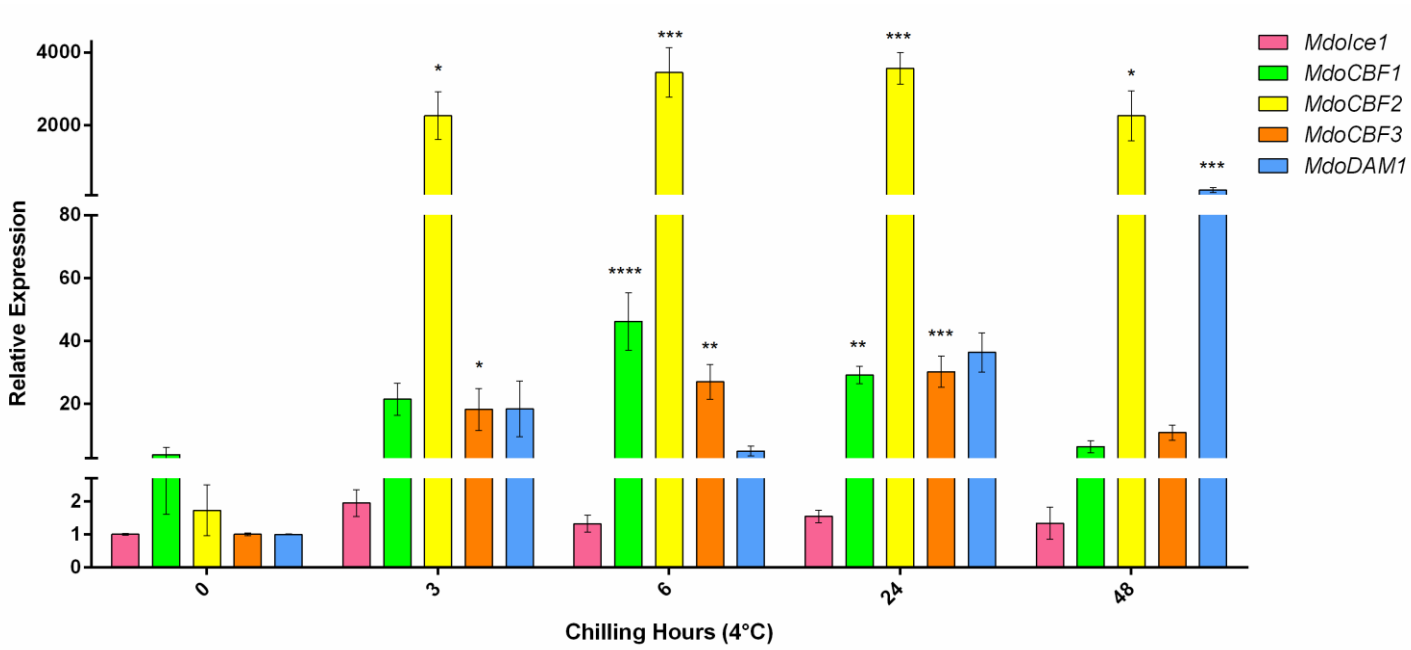

Supplement: Supplementary file 1 [file Presentation_1.zip › Supplementary Materials/Supplementary Figure 5.pdf]

# A

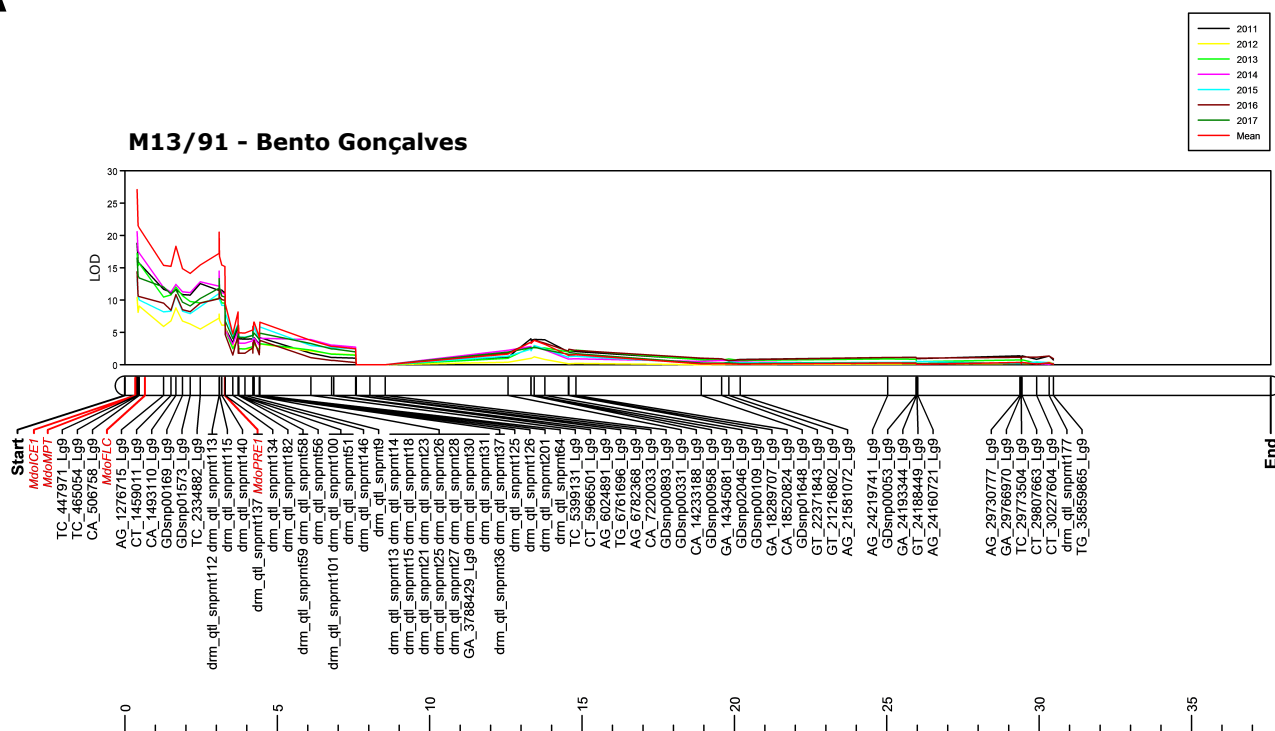

# B

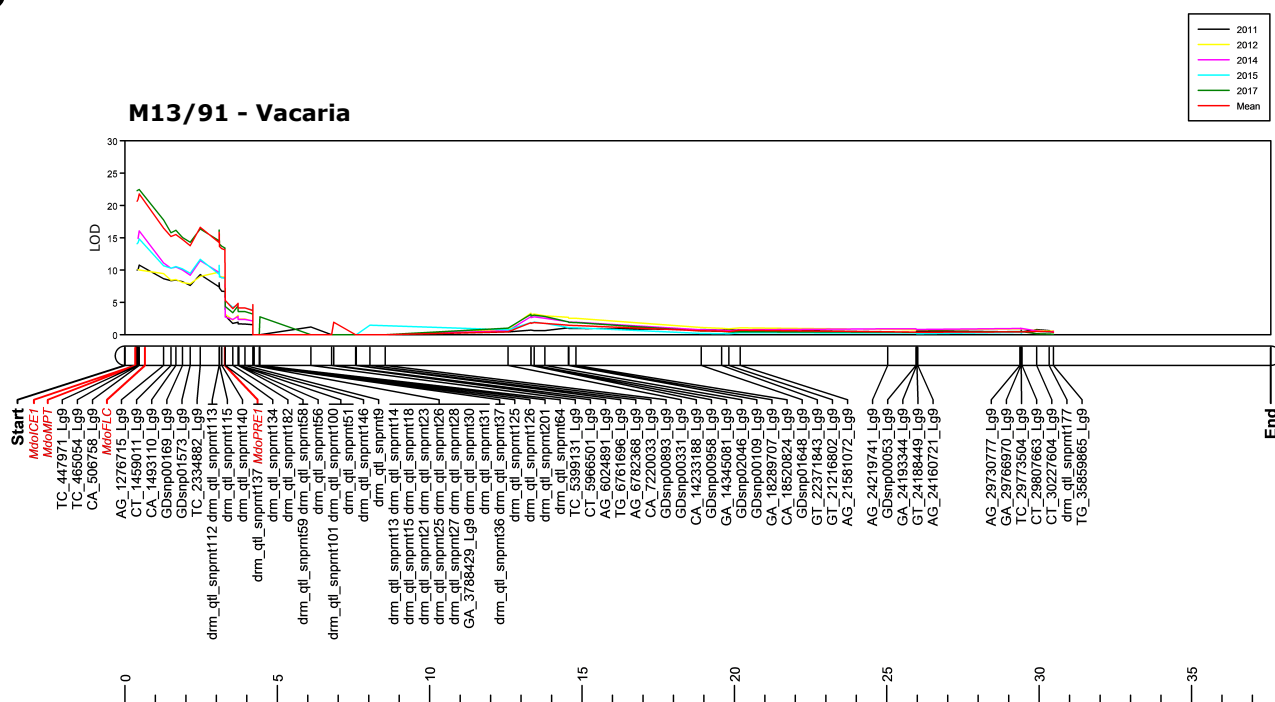

Supplement: Supplementary file 1 [file Presentation_1.zip › Supplementary Materials/Supplementary Figure 6.pdf]
